# Application of three-dimensional printing in cardiovascular diseases: a bibliometric analysis

**DOI:** 10.1097/JS9.0000000000000868

**Published:** 2023-11-03

**Authors:** Xin Zhang, Kang Yi, Jian-Guo Xu, Wen-Xin Wang, Cheng-Fei Liu, Xiao-Long He, Fan-Ning Wang, Guo-Lei Zhou, Tao You

**Affiliations:** a The First School of Clinical Medicine of Gansu University of Chinese Medicine; b Gansu International Scientific and Technological Cooperation Base of Diagnosis and Treatment of Congenital Heart Disease; c Department of Cardiovascular Surgery, Gansu Provincial Hospital; d Evidence-Based Medicine Center, School of BasicMedical Sciences, Lanzhou University; e The First Clinical Medical College of Lanzhou University, Lanzhou, People's Republic of China

**Keywords:** bibliometric analysis, cardiovascular diseases, citation analysis, three-dimensional (3D) printing

## Abstract

**Aim:**

This paper aimed to explore the application of three-dimensional (3D) printing in cardiovascular diseases, to reach an insight in this field and prospect the future trend.

**Methods:**

The articles were selected from the Web of Science Core Collection database. Excel 2019, VOSviewer 1.6.16, and CiteSpace 6.1.R6 were used to analyze the information.

**Results:**

A total of 467 papers of 3D printing in cardiovascular diseases were identified, and the first included literature appeared in 2000. A total of 692 institutions from 52 countries participated in the relevant research, while the United States of America contributed to 160 articles and were in a leading position. The most productive institution was Curtin University , and Zhonghua Sun who has posted the most articles (*n*=8) was also from there. The Frontiers in Cardiovascular Medicine published most papers (*n*=25). The Journal of Thoracic and Cardiovascular Surgery coveted the most citations (*n*=520). Related topics of frontiers will still focus on congenital heart disease, valvular heart disease, and left atrial appendage closure.

**Conclusions:**

The authors summarized the publication information of the application of 3D printing in cardiovascular diseases related literature from 2000 to 2023, including country and institution of origin, authors, and publication journal. This study can reflect the current hotspots and novel directions for the application of 3D printing in cardiovascular diseases.

## Introduction

Highlights

- This study is the first bibliometric analysis of three-dimensional (3D) printing in the field of cardiovascular diseases.
- There is a significant increase in the number of publications of 3D printing in the field of cardiovascular diseases research during the past 10 years.
- The USA was the dominant country regarding articles and researchers in 3D printing in the field of cardiovascular diseases.

Three-dimensional (3D) printing also known as additive manufacturing or as rapid prototyping refers to a production technique that allows produce prototypes or final products by stereoscopically stacking discrete materials under computer control^1^. The technology is used to convert digital objects into physical models, providing not only an improved visualization of anatomy, but also a tactile experience compared to 3D imaging^2^. In this way, a physical model can be quickly and effectively optimized. In addition, 3D printing technologies usually involve construction via layer-by-layer deposition, curing or fusion of material to create objects, as opposed to casting or traditional subtractive methods of manufacture such as machining and forging^3^. 3D printing technology has been available for about 30 years^4^. Currently, as 3D printing moved from other technology sectors into medicine, medical 3D printing also has been steadily rising particularly in surgical practice and in translational research^2,5^.

3D printing has been rapidly applied in the field of medical in recent 20 years^6^. Traditionally, this technique has been widely used in plastic surgery, orthopaedics, and dentistry^7,8^. However, the use of 3D printing technology in cardiovascular diseases is still a relatively new field^9^. In recent years, there has been a growing interest in the use of 3D printing technology in cardiovascular diseases. Anatomic relationships of cardiovascular structures are variable and complex. For these procedures to be successful, a comprehensive knowledge of patient-specific anatomy is crucial^10^. 3D printing provides a facile way of understanding complex anatomic relationships, which can complement other imaging modalities^5^. To date, 3D printing has been widely used in the treatment of heart diseases, such as congenital heart disease (CHD), valvular diseases, and hypertrophic cardiomyopathy. In cardiovascular surgery, 3D printing can provide improved visualization of the anatomical details and guide precision operations, particularly in procedural planning, communication with patients and medical education^6^.

Bibliometrics is the quantitative analysis of research publications. It was first defined in 1969 and gained widespread acknowledgement by the academia^11^. Bibliometrics can be used to understand key areas of research and to predict future research directions. As clinical research in 3D printing in cardiovascular disease is growing, and bibliometric analysis of this field may provide direction for research clinical questions and raise awareness of research trends. Therefore, it is necessary to conduct a bibliometric analysis on the application of 3D printing in cardiovascular diseases^12^. Through the analysis, the research status, hotspots and future research trend of 3D printing in cardiovascular disease fields can be determined.

## Methods

### Data source and literature inclusion criteria

We derived the data from the Web of Sciences Core Collection (WOSCC) database in 15 September 2023. WOS is one of the most widely used databases in academics, which is owned by the company Thomson & Reuters Corporation, and provides many leading journals available and detailed information about publications around the world^13^. WOS was chosen as the primary database for this study because of its comprehensive coverage of multiple academic journals and its frequent use by researchers. In addition, WOS provides the most comprehensive and reliable bibliometric analysis data compared to other databases such as Scopus, Medline and PubMed^14^. For example, data on co-cited references and co-cited journals. We searched the WOS to identify all studies relating to application of 3D printing in cardiovascular disease. The search strategy used in the fields of title, abstract, and keywords were: 3D printing, Three-dimensional printing, rapid prototyping, stereolithography, additive manufacturing, coronary, heart disease, heart attack, coronary disease, coronary event, myocardia, congenital heart disease, congenital heart defect, heart valvular disease, aortic valve, mitral valve, tricuspid valve, heart abnormal, heart malform. No time limitations were implemented nevertheless the language of literature was limited to English.

The retrieved articles from the databases were exported to EndNote X8 (Thomson Reuters (Scientific) LLC) for further categorization. Two independent reviewers (Z.X. and Y.K.) reviewed the literature for relevant articles by reading the titles and abstracts, and full texts if necessary, so that all literature of application of 3D printing in cardiovascular disease were included as much as possible. Discrepancies in the process were resolved by discussion between the two authors until they reached agreement. At length a list is output through the EndNote X8.

Publication types were limited to articles and reviews only, and meeting abstracts, editorial materials, letters, news reports and book reviews were excluded.

### Data collection and statistical analysis

The final list was exported to Microsoft Excel 2019 (Microsoft, Redmond, www. Microsoft. com) spreadsheets and the following data on each publication were extracted from the list: authors, titles, sources of journal, keywords, impact factor (IF) of journal, citations, references, institutions of authors, country of authors, co-citation authors, and co-citation journals. The overall design of this study referred to several previous bibliometric literatures. The IF of journals was obtained from the 2023 Journal Citation Reports.

### Visualization and network mapping

VOSviewer is a visual tool, it is used to make science mapping analysis of publications in the journal, because it has powerful user graphic-interface and mapping visual capability^15^. VOSviewer (1.6.16) was used to extract key information, to be specific, for the high-frequency fields such as countries, institutions and keywords^16^. VOSviewer graphically represents them through bibliometric analysis in an easy-to-understand manner. We make a network map for visual analysis, and furthermore analyze the potential clusters can identify a research field trend and measure the proximity degree. The interpretation of a visual network map based on four characteristics: size, colours, distance, and connecting line thickness. A node represents a specific term, such as country, author, or keyword, larger nodes mean higher frequency, and smaller nodes mean lower frequency. A colour represents a term, in cluster analysis, different colours represent different sub-clusters or classifications in this field. The distance between the nodes represents a relevance, and nodes that were close to each other indicated high relevance. The thickness of connecting lines represents the strength of collaboration between the nodes^17^. Before importing in VOSviewer to generate maps, data were normalized. We standardized different expressions about the same author or keyword into the uniform expression to reduce the bias in data analysis, which was completed artificially by the authors^18^. CiteSpace software was developed by Dr. Chaomei Chen who is a scholar at Drexel University, USA. The software uses Java to conduct visual analyses of scientific references. CiteSpace is one of the most sought-after bibliometric tools for investigating the evolution of a topic, and it is usually used to detect bursts^19^. CiteSpace 6.1.R6 was used in this study to detect bursts for co-occurrence items, such as authors, institutions, keywords, and co-cited references. Bursts are defined as a characteristic, which are cited frequently over a period of time. Dual-map overlay of journals was also created by CiteSpace^20^.

## Results

### Annual publications

A total of 1326 publications were retrieved from WOS Core Collection database. 467 related articles were finally identified including 117 reviews and 351 original articles, while H-index was 57^21^. Among the documents included, the first literature about application of 3D printing in cardiovascular disease was published in 2000^22^. Of the 467 articles, 24 (5%) were published between 2000 and 2010, 253 (54.2%) were published between 2010 and 2020, and 190 (40.8%) were published between 2021 and 2023. The year with the highest total number of articles was 2022.

### Countries/Institutions collaboration

**Figure 1:**
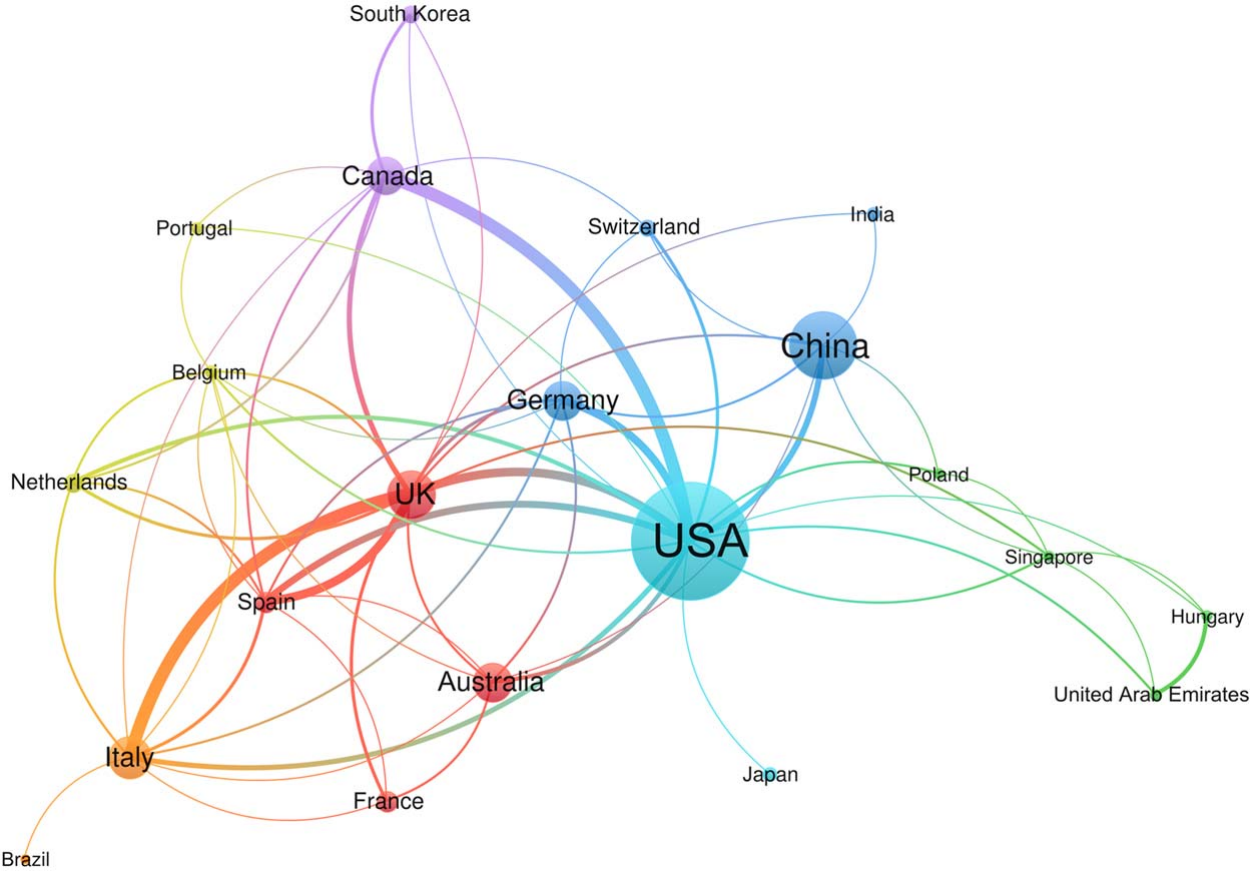
Network map of cooperation between countries of application of three-dimensional printing in cardiovascular disease.

According to the search results, there are a total of 692 institutions came from 52 countries. We have listed the top 10 countries and institutions in terms of publications (Table 1 and Table 2). The United States of America (USA) has the largest number of publications contributed to 160 articles and the China ranks second (*n*=75), followed by The United Kingdom of Great Britain and Northern Ireland (*n*=47). Curtin University published the largest number of articles, which had published 20 papers in this field, followed by University of Toronto (*n*=12). International collaboration was analyzed based on country or institution using the VOSviewer. Figure 1 presents a visualization map of collaboration between different countries. According to the collaboration of the countries which showed that USA, China, UK, Germany, and Australia maintained intimate cooperation with other countries. The thickness of connecting lines represents the strength of collaboration between the nodes. The analysis found that the USA had the strongest collaboration network, represented by a total link strength of 6416, followed by the China (total link strength 1019), and UK (total link strength was 1173). According to the analysis of the number of papers issued by 692 institutions, 502 (72.54%) institutions only participated in publishing one study, 150 (20.23%) institutions published two to three studies, 22 (3.18%) institutions published four studies, and there were 28 (4.05%) institutions published more than four studies.

**Table 1:** The top 10 countries contributed to publications in application of 3D printing in cardiovascular diseases.

Rank	Country	*n*	Citations
1	USA	160	6416
2	China	75	1019
3	UK	47	1173
4	Italy	40	773
5	Germany	35	1309
6	Australia	35	777
7	Canada	34	1424
8	France	15	540
9	Spain	15	464
10	Netherlands	13	297

3D, three dimensional.

**Table 2:** The top 10 countries and institutions contributed to publications in application of 3D printing in cardiovascular diseases.

Rank	Institution	*n*	Citations
1	Curtin University (Australia)	20	316
2	University of Toronto (Canada)	12	564
3	King’s College London (UK)	11	343
4	University of Bristol (UK)	10	224
5	University of College London (UK)	9	408
6	University of Ottawa (Canada)	8	556
7	Harvard Medical School (USA)	8	400
8	Polytechnic University of Milan (Italy)	8	225
9	Technical University of Munich (Germany)	7	320
10	Columbia University in the City of New York (USA)	7	99

3D, three dimensional.

### Authorship collaboration

**Figure 2:**
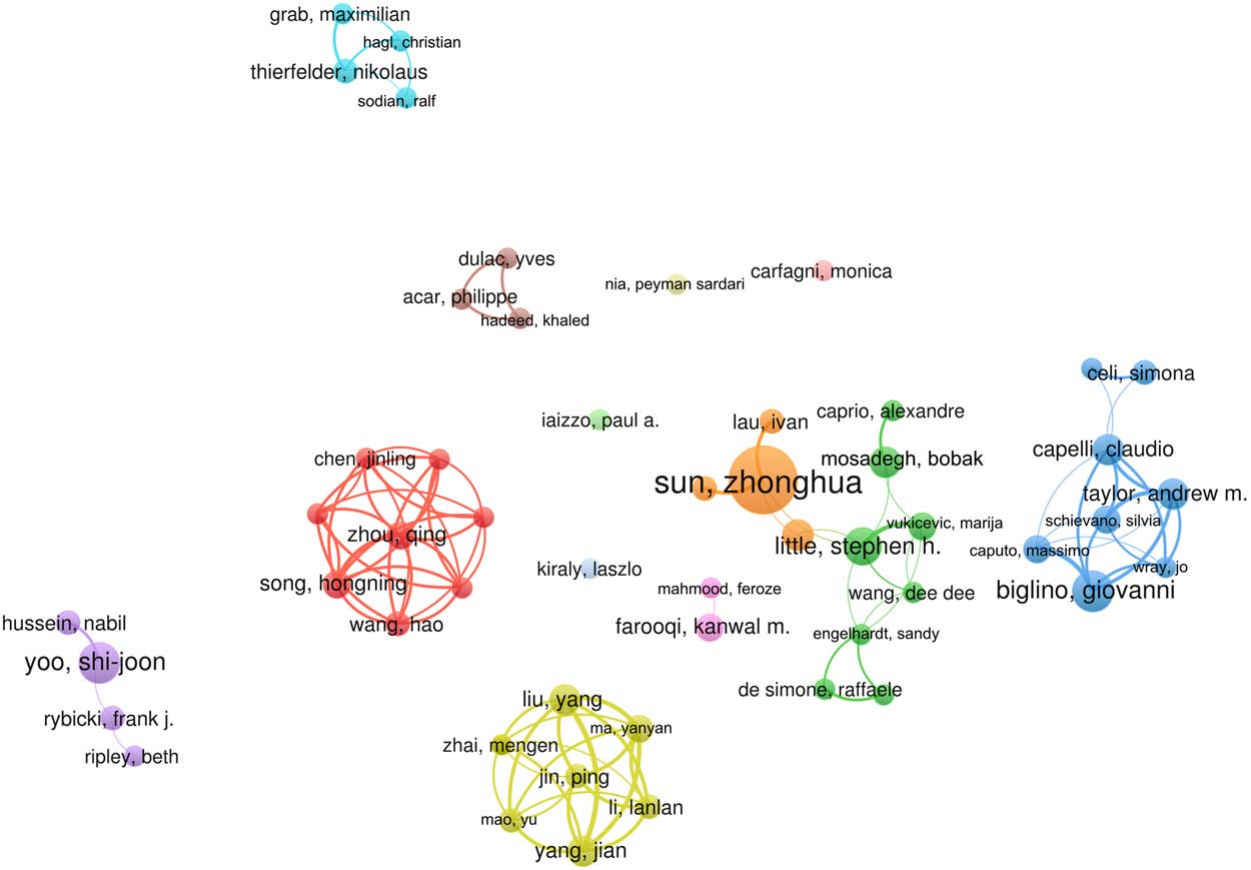
Network map of cooperation between authors of application of three-dimensional printing in cardiovascular diseases.

A total of 2457 authors participated in the field researches, and 2106 (85.71%) authors with a minimum of one study. In addition, 232 authors published two studies, 67 authors published three studies, 25 authors published four studies, and 27 authors published five to 20 studies. Zhonghua Sun ranked first among all authors, and could be considered as the most productive, who published 20 studies with a total of 316 citations, followed by Shi-Joon Yoo(documents=10, citations=656), and Giovanni Biglino (documents=10, citations=223) (Table 3). Figure 2 showed the network cooperation among 52 authors with a minimum number of documents of four (including four). A total of nine cooperative author groups were detected, and top 10 authors were from five clusters, of which four from a same cluster.

**Table 3:** The top 10 authors in application of 3D printing in cardiovascular diseases.

Author	Institution	*n*	Citations
Zhonghua Sun	Curtin University (Australia )	20	316
Shi-Joon Yoo	University of Toronto, Hosp Sick Children (Canada)	10	656
Giovanni Biglino	Great Ormond Street Hospital for Children (UK) Bristol Medical School (UK)	10	223
Stephen H. Little	Houston Methodist DeBakey Heart and Vascular Center (USA)	9	545
Andrew M Taylor	Department of Food Science and Human Nutrition, University of Illinois (USA )	7	391
Bobak Mosadegh	Department of Biomedical Engineering, University of Michigan (USA )	7	363
Israel Valverde	Paediatric Cardiology Unit, Hospital Virgen Del Rocio, Seville (Spain)	7	242
Capelli Claudio	Institute of Cardiovascular Science, University College London (UK)	7	238
Jian Yang	Department of Cardiovascular Surgery, Xijing Hospital, Fourth Military Medical University (China)	7	12
Yang Liu	Department of Cardiovascular Surgery, Xijing Hospital, Fourth Military Medical University (China)	7	12

3D, three dimensional.

### Journals and co-cited journals

The retrieved data were published in 236 different journals. Among them, 146 (61.86%) journals published only one article, 70 (29.66%) journals published two-four studies, and 20 (8.47%) journals published more than five (including five) studies. Table 4 lists the top 10 journals and co-cited journals. The Frontiers in Cardiovascular Medicine published the largest number of papers (25 publications), followed by the Interactive Cardiovascular and Thoracic Surgery (11 publications). The top 10 most productive journals published 93 of the total number of publications in the field. The average IF with the top 10 journals was 3.2378. In the citation analysis, 32 journals with a total number of citations of more than 100. The top five most cited journals were Journal of Thoracic and Cardiovascular Surgery (*n*=520), Annals of Thoracic Surgery (*n*=498), Circulation (*n*=477), Journal of the American College of Cardiology (*n*=466), and Biomaterials (*n*=413). Journal of Thoracic and Cardiovascular Surgery is in a leading position about the number of citations.

**Table 4:** The top 10 journals and co-cited journals in application of 3D printing in cardiovascular diseases.

Rank	Journal	*n*	Citations	Country	IF	Co-Cited Journal	Citations	Country	IF
1	Frontiers in Cardiovascular Medicine	25	44	Switzerland	3.6	Journal of Thoracic and Cardiovascular Surgery	520	USA	6
2	Interactive Cardiovascular and Thoracic Surgery	11	123	UK	1.978	Annals of Thoracic Surgery	498	USA	4.6
3	Catheterization and Cardiovascular Interventions	10	294	USA	2.3	Circulation	477	USA	37.8
4	Current Cardiology Reports	8	72	USA	3.7	Journal of the American College of Cardiology	466	USA	24
5	European Journal of Cardio-Thoracic Surgery	7	484	Netherlands	3.4	Biomaterials	413	Netherlands	14
6	Frontiers in Pediatrics	7	147	Switzerland	2.6	Jacc-Cardiovascular Imaging	371	USA	14
7	3D Printing in Medicine	7	52	UK	3.7	European Journal of Cardio-Thoracic Surgery	333	Netherlands	3.4
8	Annals of Thoracic Surgery	6	303	USA	4.6	Jacc-Cardiovascular Interventions	277	USA	11.3
9	Biomolecules	6	85	Switzerland	5.5	European Heart Journal	209	UK	39.3
10	Cardiology in the Young	6	65	USA	1	Acta Biomaterialia	190	UK	9.7

3D, three dimensional.

**Figure 3:**
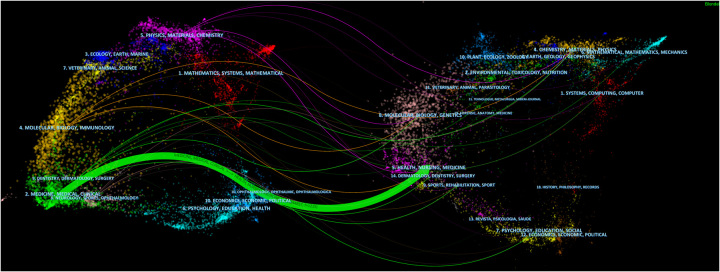
The dual-map overlay of journals related to application of three-dimensional printing in cardiovascular diseases.

In order to analyze the distribution of published journals and cited journals more specifically, we carried out the double-map overlaying by visual analysis method (Fig. 3). The map on the left represented the citing journal. The map on the right represented the cited journal. The label represented the subject covered by the journal. Coloured curves represent paths of references, originating from the citing map on the left and pointing to the cited map on the right. There was 1 main citation path in the current map. The path referred that these papers published in medicine/medical/ clinical, and the most cited papers were mainly published in health/ nursing/medicine.

### Keywords mapping

A total of 1605 keywords were extracted and the total frequency of occurrence was 4276. The top 20 keywords about application of 3D printing in cardiovascular diseases were listed in Table 5. We used CiteSpace to analyze keywords bursts to monitor the dynamic changes about research in the study. At present the top 70 key words with the highest citation bursts were the indicators of research frontier topics. The early keywords (from 2000 to 2015) monitored with a burst were rapid prototyping, computed tomography (CT), and echocardiography; but between 2016 and 2023, the key words with the strongest citation bursts were surgery, fibrillation, mitral valve, congenital heart defect, heart valve, education, surgical simulation, and preoperative planning. This result indicates that medical education, surgical planning, and simulation have become the research development trend and focus on this field of research. (Appendix Figure A2, Supplemental Digital Content 1, http://links.lww.com/JS9/B282)

**Table 5:** The top 20 keywords in application of 3D printing in cardiovascular diseases.

Rank	Keyword	*n*	Rank	Keyword	*n*
1	3D printing	333	11	Heart	32
2	3D models	127	12	Implantation	31
3	Congenital heart disease	116	13	Preoperative planning	30
4	Simulation	97	14	Cardiovascular disease	29
5	Cardiovascular surgery	88	15	Management	24
6	Medical education	57	16	Outcomes	24
7	CT	49	17	Technology	24
8	Repair	41	18	Atrial fibrillation	23
9	Echocardiography	36	19	Replacement	23
10	Mitral valve	35	20	Aortic valve	22

3D, three dimensional; CT, computed tomography.

**Figure 4:**
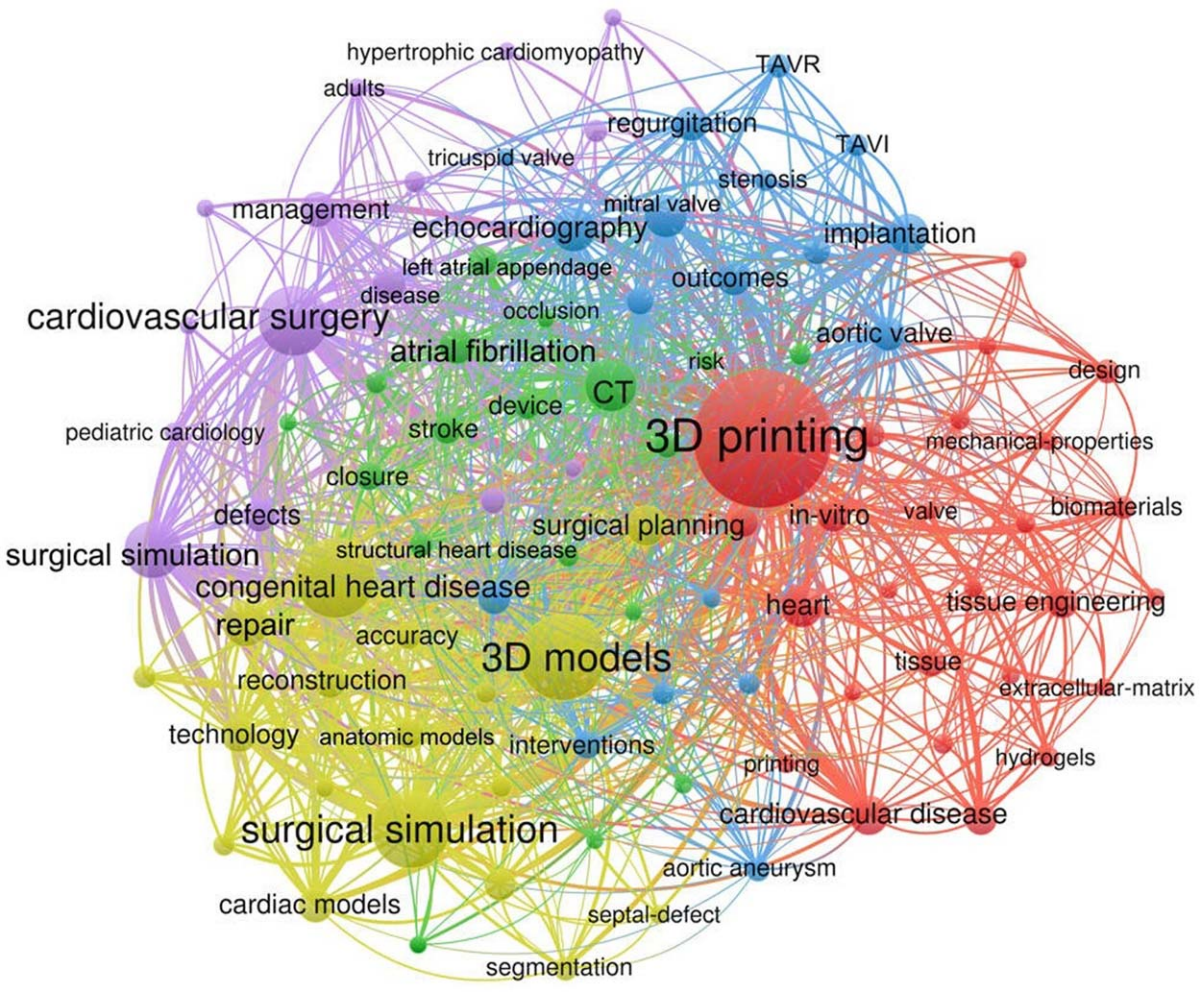
The network map of keywords in application of 3D printing in cardiovascular diseases. 3D, three dimensional.

Cluster analysis for keywords with six or more times were conducted, and five clusters were detected. The keywords with high frequency can accurately reveal the main topic of a field. In cluster Ⅰ with blue colour, the keywords with a high occurrence were mitral valve, paravalvular leak, TAVR etc. In cluster Ⅱ with purple colour, the keywords with a high occurrence were medical education, paediatric cardiology, etc. In cluster Ⅲ with green colour, the keywords with a high occurrence were occlusion, atrial fibrillation, left atrial appendage, occlusion, etc. In cluster Ⅳ with yellow colour, the keywords with a high occurrence were congenital heart disease, cardiac models, surgical planning, surgical simulation, etc. In cluster V with red colour, the keywords with a high occurrence were cardiovascular disease, bioprinting, biomedical application, etc. Some methodological keywords appearing in the cluster analysis centre should not be simply classified into any cluster, such as 3D printing, CT, 3D models etc (Fig. 4).

### Co-cited references and bursts detection

Co-cited references refer to documents that have been co-cited by the 467 studies included in the analysis. The top 10 co-cited references were listed in Table 6. These highly co-cited studies were all reviews. All of Co-cited references is related to the field by theory, applications, research, and development. Three references were co-cited more than 60 times. These articles summarized that 3D printing was applied in cardiac surgery. The most co-cited literature was published in JACC Cardiovasc Imaging.

**Table 6:** Top 10 co-cited references related to application of 3D printing in cardiovascular disease.

Rank	Co-cited reference	Co-citation
1	Vukicevic M, Mosadegh B, Min JK, Little SH. Cardiac 3D Printing and its Future Directions. JACC Cardiovasc Imaging. 2017 Feb;10(2):171-184.	81
2	Ripley, B., Kelil, T., Cheezum, M. K., Goncalves, A., Di Carli, M. F., Rybicki, F. J., Steigner, M., Mitsouras, D., & Blankstein, R. (2016). 3D printing based on cardiac CT assists anatomic visualization prior to transcatheter aortic valve replacement. Journal of cardiovascular computed tomography, 10(1), 28–36.	60
3	Schmauss, D., Haeberle, S., Hagl, C., & Sodian, R. (2015). Three-dimensional printing in cardiac surgery and interventional cardiology: a single-centre experience. European journal of cardio-thoracic surgery : official journal of the European Association for Cardio-thoracic Surgery, 47(6), 1044–1052.	60
4	Valverde, I., Gomez-Ciriza, G., Hussain, T., Suarez-Mejias, C., Velasco-Forte, M. N., Byrne, N., Ordoñez, A., Gonzalez-Calle, A., Anderson, D., Hazekamp, M. G., Roest, A. A. W., Rivas-Gonzalez, J., Uribe, S., El-Rassi, I., Simpson, J., Miller, O., Ruiz, E., Zabala, I., Mendez, A., Manso, B., … Hosseinpour, A. R. (2017). Three-dimensional printed models for surgical planning of complex congenital heart defects: an international multicentre study. European journal of cardio-thoracic surgery : official journal of the European Association for Cardio-thoracic Surgery, 52(6), 1139–1148	59
5	Costello, J. P., Olivieri, L. J., Su, L., Krieger, A., Alfares, F., Thabit, O., Marshall, M. B., Yoo, S. J., Kim, P. C., Jonas, R. A., & Nath, D. S. (2015). Incorporating three-dimensional printing into a simulation-based congenital heart disease and critical care training curriculum for resident physicians. Congenital heart disease, 10(2), 185–190	57
6	Biglino, G., Capelli, C., Wray, J., Schievano, S., Leaver, L. K., Khambadkone, S., Giardini, A., Derrick, G., Jones, A., & Taylor, A. M. (2015). 3D-manufactured patient-specific models of congenital heart defects for communication in clinical practice: feasibility and acceptability. BMJ open, 5(4), e007165	56
7	Olivieri, L. J., Krieger, A., Loke, Y. H., Nath, D. S., Kim, P. C., & Sable, C. A. (2015). Three-dimensional printing of intracardiac defects from three-dimensional echocardiographic images: feasibility and relative accuracy. Journal of the American Society of Echocardiography : official publication of the American Society of Echocardiography, 28(4), 392–397	54
8	Giannopoulos, A. A., Mitsouras, D., Yoo, S. J., Liu, P. P., Chatzizisis, Y. S., & Rybicki, F. J. (2016). Applications of 3D printing in cardiovascular diseases. Nature reviews. Cardiology, 13(12), 701–718	49
9	Schmauss, D., Schmitz, C., Bigdeli, A. K., Weber, S., Gerber, N., Beiras-Fernandez, A., Schwarz, F., Becker, C., Kupatt, C., & Sodian, R. (2012). Three-dimensional printing of models for preoperative planning and simulation of transcatheter valve replacement. The Annals of thoracic surgery, 93(2), e31–e33	49
10	Costello, J. P., Olivieri, L. J., Krieger, A., Thabit, O., Marshall, M. B., Yoo, S. J., Kim, P. C., Jonas, R. A., & Nath, D. S. (2014). Utilizing Three-Dimensional Printing Technology to Assess the Feasibility of High-Fidelity Synthetic Ventricular Septal Defect Models for Simulation in Medical Education. World journal for pediatric & congenital heart surgery, 5(3), 421–426	48
10	Maragiannis, D., Jackson, M. S., Igo, S. R., Schutt, R. C., Connell, P., Grande-Allen, J., Barker, C. M., Chang, S. M., Reardon, M. J., Zoghbi, W. A., & Little, S. H. (2015). Replicating Patient-Specific Severe Aortic Valve Stenosis With Functional 3D Modeling. Circulation. Cardiovascular imaging, 8(10), e003626	48
10	Yoo, S. J., Spray, T., Austin, E. H., 3rd, Yun, T. J., & van Arsdell, G. S. (2017). Hands-on surgical training of congenital heart surgery using 3-dimensional print models. The Journal of thoracic and cardiovascular surgery, 153(6), 1530–1540	48

3D, three dimensional.

**Figure 5:**
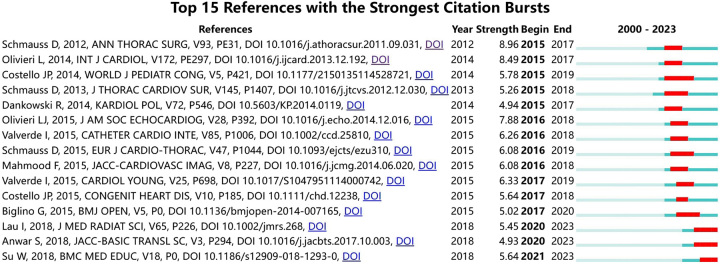
Top 15 References with the strongest citation bursts.

References with citation burst refers to documents that have been highly cited in a period of time. As shown in Figure 5, the threshold was set to top 15 in a 1-year slice in CiteSpace, strong citation bursts with a minimum duration of three year were found in 15 co-cited references. The strongest burst was detected in two papers, and their duration of high citation was three years. These two papers focused on the use of 3D-printed models in surgical planning of cardiovascular diseases. Appendix Figure A1, Supplemental Digital Content 1, http://links.lww.com/JS9/B282 showed that a strong citation burst with a minimum duration of one year was found in 25 co-cited authors. The dynamics of a field can be characterized in part by studies with citation bursts; these co-cited references and authors represented the current state of development for the application of 3D printing in cardiovascular diseases.

## Discussion

Bibliometric analysis is a tool to quantify the characteristics and scholarly impact of citation classics. In this study, the literature on application of 3D printing in cardiovascular disease in the WOSCC database was bibliometriccally analyzed by using Citespace and VOSviewer. This bibliometric study aimed to analyze the current status, hotspots and trends about application of 3D printing in cardiovascular diseases.

Mapping the authors collaboration and countries/regions cooperation networks is an essential class of scientific social-networks, which is used for showing the key structure of scientific collaboration and the position of countries/regions. Developed countries may have a better impact on research productivity and quality, and they can allocate better economics and spending to scientific research. The Table 1 showed the USA has the largest number of publications in the field, followed by the China, UK , and Italy. China is also the only developing country in the top 10 countries. Network cluster analysis showed sufficient cooperation between countries and institutions. There were seven countries published more than 30 studies. In addition, UK had the largest number of institutions. This finding indicated that European and North American countries were still the main sources of clinical research publications in this field. However, publications from China in the field have begun to increase and are expected to make important contributions to this field in the future.

Among the top 10 authors, the author who published the most studies was Zhonghua Sun from the Curtin University. In the cluster analysis, four of the top 10 authors came from the same cluster. The other six authors have their own relatively fixed cooperative groups. Cluster analysis revealed that only Sun, Zhonghua ‘s team and Stephen H. Little’s team have cooperated, the rest of the clusters are independent of each other in the Fig. 2. The yellow cluster represent a large group of authors, which indicates a wide range of cooperation among authors in this field. Although there is only two author with outstanding a number of published papers, the authors in these cluster still show good potential and prospects, as well as the diversity of author groups. It can be predicted that more high-yield authors will appear in these cluster in the future.

Journal analysis found that there were 146 journals published one study in this field, which accounted for 61.86% of the total publications. The top three journals published 19.49% of studies in this field. The *Frontiers in Cardiovascular Medicine* published the most studies. Next came the *Interactive Cardiovascular and Thoracic Surgery* and *Catheterization and Cardiovascular Interventions*. Among the top 10 journals, four are from USA, three from Switzerland, two from UK and one from Netherlands. The IF of these journals can also reflect the importance and priority of the field.

The top one co-cited article entitled Cardiac 3D Printing and its Future Directions, it came from JACC-Cardiovascular Imaging. This article summarized that 3D printing is a new tool that is challenging how we image, plan, and carry out cardiovascular interventions. The most researchers obtained their citations from a few main journals in their respective field of expertise. When the researchers deviate from these core journals, their citation frequency and impact were weakened. Consequently, this tendency led to a large percentage of citations stemming from a few core journals^9^.

A map of keywords can reflect hot topics of research. In addition, keywords can represent the core point of an article, keywords analysis can be used for detecting research topics, as well as for monitoring the research frontier transitions of a certain knowledge domain. Through the analysis of the high-frequency keywords in the literature in this field, we can find from the top 20 keywords listed in Table 5, the most frequent key word was 3D printing followed by 3D models and congenital heart disease. In keyword network clustering analysis, the five clusters were about (I) valvular heart disease; (II) medical education; (III) left atrial appendage occlusion; (IV) congenital heart disease and (V) biomedical application

With further maturation of 3D printing, it is being incorporated into more mainstream procedures such as transcatheter aortic valve replacement (TAVR)^23^. The first case of TAVR was reported in 2002 by Cribier *et al.*^8^. This type of operation does not require cardiopulmonary bypass and recovery is rapid in postoperative^8,23,24^. Since its launch, TAVR has been increasingly used to treat severe aortic valve disease and has benefited more than 500 000 patients. 3D printing can be applied to the implementation of TAVR, which includes optimizing choice of patient, prediction the possibility of conduction block, coronary artery occlusion, paravalvular leakage^23,24^. In particular, paravalvular leakage is more common than surgical replacement and is associated with increased mortality^25^. Therefore, 3D models of the aortic root and valve can be printed in order that provide a better understanding of the anatomy and the preparation of the TAVR procedure^26^. Moreover, the experience of aortic root printing is gradually emerging, which may improve the success and safety of TAVR^2,26^. Similarly, there was study has shown that 3D printing is applied to mitral valve disease. Mitral regurgitation (MR) is the second most frequent clinically relevant valve diseases, and its prevalence increases with the age of patients^27–29^. Although surgical repair or replacement of the mitral valve is the primary treatment for severe MR, surgery is too risky for elderly patients or those with significant comorbidities. Therefore, more and more transcatheter mitral valve replacement (TMVR) is being considered for these patients^27^. However, transcatheter mitral valve replacement remains challenging because it is difficult to predict device interactions with complex native anatomy. Therefore, 3D printing methodology might be a powerful aid to such complex procedures. The first report of preprocedural 3D printing to test transcatheter mitral valve intervention was published in 2016^30^. The rate of transfer from repair to replacement, along with surgery time, could be reduced with the aid of the 3D-printed models. In addition, a recent study shown that 3D printing is applied to percutaneous interventions on the tricuspid valve disease^31^. There are already several reports summarizing how 3D print modelling has been used to facilitate successful tricuspid valve interventions. For instance, Harb and colleagues used the 3D-printed models for the estimation of tricuspid morphology, to enhance the preprocedural planning of percutaneous interventions and 3D-printed models be used to select the appropriate size of the device and test the implantation^32^. In addition, Spring and colleagues used 3D-printed models to guide tricuspid valve-in-valve replacement^32,33^.

Cluster analysis also found the enthusiasm of researchers to use 3D printing in medical education related research, which found 3D printing can deal with a variety of clinical challenges, including patient education and medical professionals training. Meanwhile, the early adoption of physical 3D printing technology with patient communication has improved the understanding and feedback of operation informed consent^31^. Moreover, preoperative simulation for trainee surgeons of paramount importance in order to minimalize any potential risk to patient safety and to increase their confidence and technical skills associated with the procedure^34–36^. At the same time, 3D printing cardiac models also are used in medical student education. The most common modes of medical education for heart disease rely heavily on 2D imaging. 3D printing technology allows for the creation of physical cardiac models that can be used for teaching medical student. Such models can be instructional for the teaching of medical professionals about normal and abnormal structural relationships, and even to help the lay public better understand certain structural heart conditions^37–39^.

More interestingly, 3D printing have also been widely used in left atrial appendage (LAA) occlusion. Atrial fibrillation is the most common arrhythmia encountered, and is one of the primary causes of ischaemic stroke^40^. LAA device closure is a relatively novel treatment option for patients with non-valvular AF who cannot tolerate long-term anticoagulation. 3D printing used cardiac computed tomography angiography data to construct a 3D structural image of the LAA that not only accurately shows the morphology of the LAA but also allows the measurement of the LAA size and other relevant parameters^41^. Furthermore, use of a physical 3D model has several advantages. Device fit can be immediately assessed visually. Physicians and the patients can also readily appreciate how the device will re-shape the LAA. Importantly, determination of the best landing zone can be accurately tested in the model thus avoiding errors at the time of implantation.

What’s more, 3D printing has also been applied to CHD, complex CHD, particularly. They are often associated with complex and unique geometry that can be very difficult to fully appreciate from CT, MRI or echocardiographic images^19^. As such, 3D-printed models may play a key role to provide a more comprehensive understanding and functional evaluation of various congenital heart conditions. A prior study demonstrating that 3D heart models can be used to enhance congenital cardiac critical care^42^. 3D printed to enhance surgical planning including: double-outlet right ventricle; atrial septal defect, ventricular septal defect, tetralogy of Fallot, hypoplastic left heart syndrome, transposition of the great arteries, and pulmonary vein ectopic drainage^41,42^. With the help of these models, which clearly showed the location and size of the abnormal anatomy and the spatial anatomy of the great artery and it can help shorten the time of surgery for complex congenital heart disease and reduce surgical complications^43,44^. Yoo *et al.*^45^ show that 3D cardiovascular models are superior to imaging data for preoperative surgical training and evaluation. In addition, study has shown that 3D-printed models also been applied to CHD morphology teaching, most of these 3D cardiovascular models were printed for surgical planning and simulation; unique or rare anatomy were printed for medical or patient education^46^. Traditionally, CHD morphology teaching has heavily relied on observation of pathologic specimens removed from deceased patients or at cardiac transplantation. However, pathologic specimens are rare resources, 3D-printed replicas of CHD are extremely valuable educational resources as almost all variations of congenital heart diseases can be reproduced^47,48^.

In recent years, 3D printing are increasingly used in biomedical applications. 3D bioprinting is the most challenging part of 3D printing technology. Based on 3D bioprinting, heart tissue can be printed for heart repair or replacement of diseased hearts, restoring heart function to a certain extent^49^. Due to the complexity of the internal structure of the heart, achieving the one-time formation of the human heart is still very difficult. Bejleri *et al.*^50^ printed cardiac patches, which were used to enhance the contractile activity of the heart of a person who had a heart attack or other injury event, and they could restore the normal ability of the damaged areas of the heart, thereby improving the overall function of the heart. Duan *et al.*^51^ printed cardiac aortic valves using aortic root sinus smooth muscle cells and aortic valve leaflet interstitial cells, as well as hydrogels made from alginate and gelatin. Recently, Jacqueline Bliley and colleagues reported the 3D bioprinting of a simplified model of the heart. They used hydrogel to print out a biological model of human heart tube, which has contractility, calcium activity and the ability of action potential propagation^52^. These results establish the feasibility of creating a 3D bioprinted human heart tube and serve as an initial step towards engineering more complex heart muscle structures. At present, 3D bioprinting technology has shown great advantages in the construction of in vitro hearts due to its accuracy and flexibility, and it has become a research hotspot in this field.

3D printing allows for a more precise understanding of the complex anatomy associated with various heart diseases. With the further development of 3D printing technology, the advantages of customization and visualization will help people to better understand cardiovascular diseases. It has been 20 years since the first report of 3D printing in cardiology. 3D printing has been developed for different purposes including preoperative evaluation, device development, hemodynamic simulation and medical education. However, most of the studies are case reports, not enough to draw strong conclusions about the efficacy of 3D printing on cardiovascular diseases. More research on rigorous design is needed to validate its impact on changing clinical practice^53^.

This study still has certain limitations: (1) the data were retrieved from the single database of the Web of Science Core Collection, did not include other databases, excluded non-English papers and there may be a certain bias in the sources of the included documents. (2) Part of the results were standardized manually before analysis to reduce the deviation caused by different expressions of the same concept, and such errors can only be reduced not completely eliminated. (3) Editorials and conference papers are not included, which will miss some representative publications, but this is for the sake of ensuring the formality and completeness of the included literature. (4) The total number of included literatures is small.

## Conclusion

We summarized the publication information of the application of 3D printing in cardiovascular diseases related literature from 2000 to 2023, including country, institution, author, publication journal, keyword, and co-cite reference. We then analyzed the research hotspots based on these publications. The cooperation between the author and the organization embodies good communication and cooperation and is full of vitality. Future research on diseases will still focus on congenital heart disease, valvular heart disease, and left atrial appendage closure. The trend can be predicted, such as treatment of medical education, 3D bioprinting and surgical training, which will be the hotspots and frontiers of research in the next few years.

## Ethical approval

As an article in bibliometric analysis, there is no ethical approval is required.

## Consent

Not applicable.

## Sources of funding

Medicine Research Fund Project of Gansu Provincial Hospital (18GSSY5-19), Natural Science Foundation of Gansu Province (22JR5RA655), Gansu University of Traditional Chinese Medicine Graduate Innovation and Entrepreneurship Fund (2022CX55).

## Author contribution

Conceptualization: X.Z., K.Y., T.Y. Data curation: X.Z., K.Y., W.-X.W., J.-G.X., C.-F.L., X.-L.H., F.-N.W., G.-L.Z. Formal analysis: X.Z., K.Y., T.Y. Investigation: X.Z., K.Y., W.-X.W., J.-G.X., C.-F.L., T.Y. Methodology: X.Z., K.Y., T.Y. Supervision: T.Y. Visualization: X.Z., K.Y. Writing—original draft: X.Z., K.Y. Writing—review and editing: X.Z., K.Y., W.-X.W., J.-G.X., C.-F.L., X.-L.H., F.-N.W., G.-L.Z., T.Y.

## Conflicts of interest disclosure

The authors declared that there have no conflicts of interest in this work.

## Research registration unique identifying number (UIN)

Not applicable.

## Guarantor

Xin Zhang, Kang Yi, and Tao You.

## Data statement

The data underlying this article are available in Web of Science Core Collection database.

## Provenance and peer review

Not commissioned, externally peer-reviewed.

## Supplementary Material
