# Supplementary material for: Application of three-dimensional printing in cardiovascular diseases: a bibliometric analysis
Source: Int J Surg. 2023 Nov 3;110(2):1068–78. doi: 10.1097/JS9.0000000000000868 (PMC10871659; doi:10.1097/JS9.0000000000000868)
Supplement: Supplementary file 1 [file js9-110-1068-s001.pdf]

Figure A1

Top 25 Cited Authors with the Strongest Citation Bursts

Cited Authors	Year	Strength	Begin	End
DURSO PS	2000	5.31	**2000**	2015
GILON D	2000	3.88	**2000**	2015
SODIAN R	2002	4.47	**2002**	2018
WINDER J	2007	5.53	**2007**	2015
NGAN EM	2007	4.58	**2007**	2015
NOECKER AM	2008	8.08	**2008**	2018
SCHIEVANO S	2008	6.01	**2008**	2017
MOTTL-LINK S	2008	4.2	**2008**	2017
OLIVIERI L	2015	8.51	**2015**	2017
KIM MS	2010	7.97	**2015**	2018
JACOBS STEPHAN	2010	4.45	**2015**	2017
VRANICAR M	2015	4.1	**2015**	2018
POTERUCHA JT	2016	4.35	**2016**	2018
MAHMOOD F	2016	4.32	**2016**	2018
YAN CW	2017	5.01	**2017**	2019
WANG ZM	2019	3.91	**2019**	2020
LAU I	2019	7.14	**2020**	2023
BATTEUX C	2020	4.14	**2020**	2023
ONG CS	2019	3.75	**2020**	2023
HUSSEIN N	2021	5.83	**2021**	2023
SUN ZH	2019	5.53	**2021**	2023
SU W	2019	4.7	**2021**	2023
TUNCAY V	2021	4.03	**2021**	2023
SMERLING J	2020	3.8	**2021**	2023
LEE A	2021	3.67	**2021**	2023

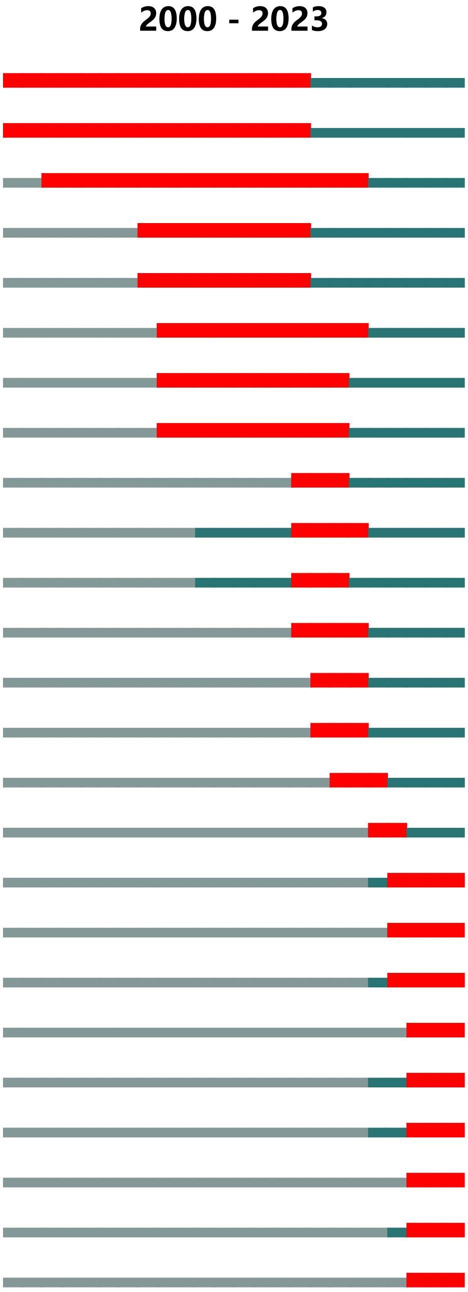

Figure A2

Top 70 Keywords with the Strongest Citation Bursts

Keywords	Year	Strength	Begin	End
ste eolithographic model	2000	1.91	**2000**	2008
tissue	2000	1.55	**2000**	2016
fused deposition modelling	2004	1.31	**2004**	2007
conduit	2004	1.27	**2004**	2018
engineered heart valve	2004	1.25	**2004**	2012
tissue engineering	2004	1.2	**2004**	2009
mr angiography	2005	1.32	**2005**	2007
dynamics	2005	1.29	**2005**	2008
rapid prototyping	2006	6.13	**2006**	2017
ct	2006	2.39	**2006**	2015
stereolithography	2006	1.15	**2006**	2012
follow up	2007	2.3	**2007**	2016
replacement	2007	1.28	**2007**	2010
anatomical model	2008	1.47	**2008**	2017
artery	2008	1.18	**2008**	2016
vascular graft	2009	1.63	**2009**	2016
construct	2009	1.35	**2009**	2012
cell	2009	1.16	**2009**	2015
echocardiography	2014	2.72	**2014**	2017
in vitro	2014	1.33	**2014**	2015
3d ultrasound	2014	1.31	**2014**	2015
mitral valve repair	2014	1.17	**2014**	2018
anatomic model	2015	2.06	**2015**	2019
3d reconstruction	2015	1.86	**2015**	2016
valve replacement	2015	1.62	**2015**	2018
3d echocardiography	2015	1.33	**2015**	2018
3d imaging	2015	1.22	**2015**	2016
3d segmentation	2015	1.22	**2015**	2016
aortic stenosis	2015	1.21	**2015**	2018
clinical application	2015	1.19	**2015**	2016
pulmonary valve implantation	2016	2.26	**2016**	2017
surgery	2000	5.39	**2017**	2018
preoperative planning	2017	2.77	**2017**	2018
fibrillation	2017	2.54	**2017**	2018
reconstruction	2000	2.54	**2017**	2018
congenital heart disease	2007	1.75	**2017**	2018

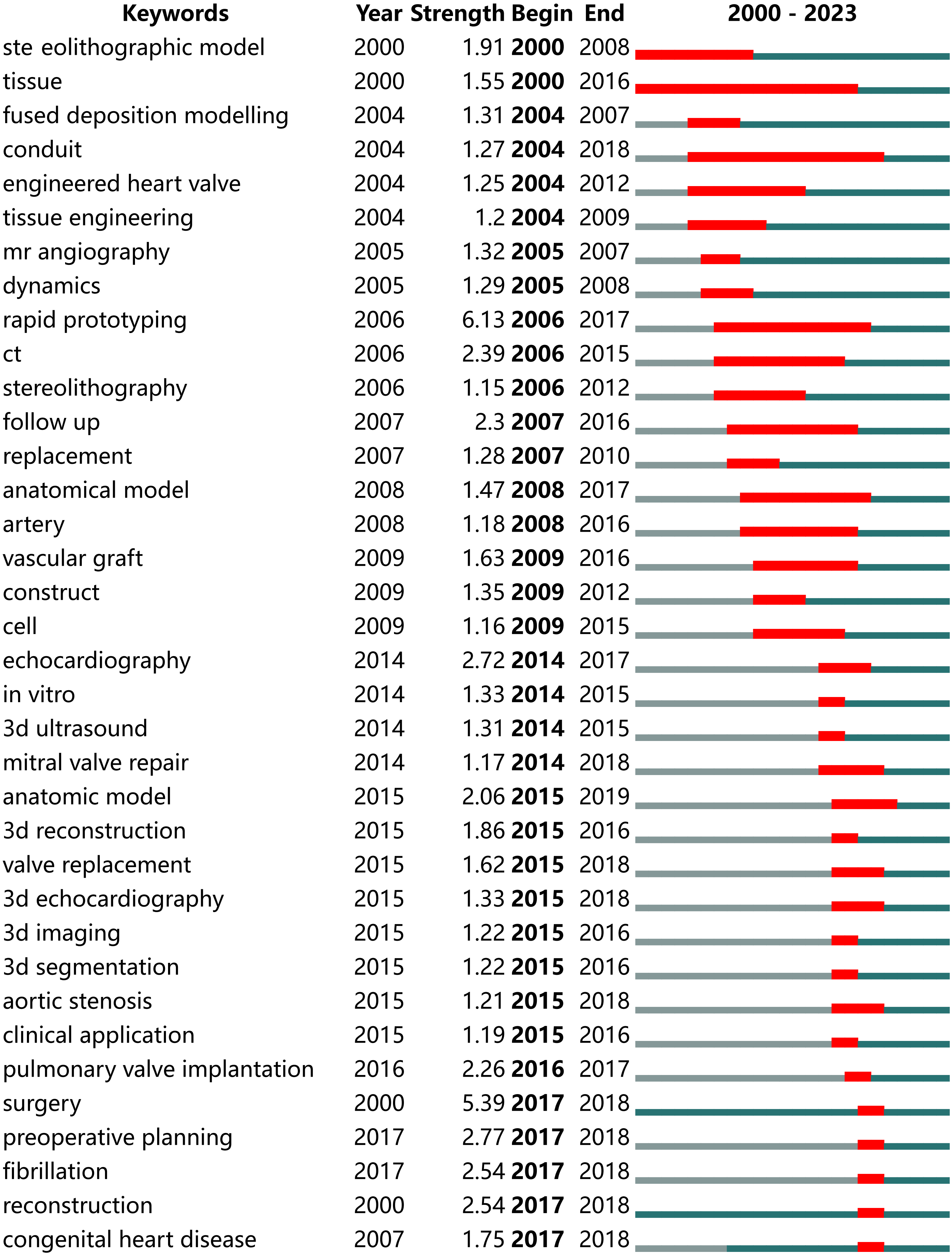

anatomy	2017	1.61	**2017**	2018
left atrial appendage	2017	1.48	**2017**	2020
stroke	2017	1.3	**2017**	2019
cardiac ct	2017	1.25	**2017**	2019
closure	2017	1.14	**2017**	2018
device	2018	3.03	**2018**	2020
surgical training	2018	2.05	**2018**	2019
septal defect	2018	2.05	**2018**	2019
transesophageal echocardiography	2018	1.9	**2018**	2019
interventional cardiology	2018	1.64	**2018**	2019
risk	2018	1.64	**2018**	2019
feasibility	2018	1.49	**2018**	2019
patient	2008	1.3	**2018**	2019
adult	2008	1.2	**2018**	2019
mitral valve	2017	4.09	**2019**	2020
heart valve	2004	2.74	**2019**	2020
defect	2000	2.17	**2019**	2021
pluripotent stem cell	2019	1.92	**2019**	2021
outlet right ventricle	2018	1.9	**2019**	2020
design	2008	1.78	**2019**	2021
matrix	2019	1.75	**2019**	2020
coronary artery	2020	2.12	**2020**	2021
atrial fibrillation	2016	1.57	**2020**	2021
case report	2020	1.27	**2020**	2023
outcm	2017	3.9	**2021**	2023
congenital heart surgery	2015	2.17	**2021**	2023
cardiovascular disease	2017	1.95	**2021**	2023
ct angiography	2021	1.7	**2021**	2023
education	2015	1.59	**2021**	2023
tricuspid regurgitation	2021	1.33	**2021**	2023
3d bioprinting	2019	1.16	**2021**	2023
cardiothoracic surgery	2018	1.16	**2021**	2023
surgical planning	2018	1.15	**2021**	2023
regurgitation	2015	1.14	**2021**	2023
